# Subjective and Objective Stress in Inflammatory Bowel Disease

**DOI:** 10.3390/life16060914

**Published:** 2026-05-28

**Authors:** Susanna Jäghult, Susana Soto Villagran, Anna Kalpouzou, Maria Kumlin, Marie Svedberg

**Affiliations:** 1 Department of Clinical Science and Education, Södersjukhuset, Karolinska Institutet, SE-118 82 Stockholm, Sweden; 2 Capio Gastro Center, SE-11486 Stockholm, Sweden; 3 Department of Health Promoting Science, Sophiahemmet University, SE-11428 Stockholm, Swedenmaria.kumlin@shh.se (M.K.); marie.svedberg@shh.se (M.S.); 4 Division of Physiological Chemistry II, Department of Medical Biochemistry and Biophysics, Karolinska Institutet, Biomedicum 9A, SE-17165 Stockholm, Sweden

**Keywords:** inflammatory bowel disease, perceived stress, objective stress, cortisol, perceived stress scale, short health scale

## Abstract

Background: Stress may have an impact on the course of inflammatory bowel disease (IBD), but evidence is still lacking regarding a potential association between perceived (subjective) stress and objectively measured stress and whether patients’ levels of stress in relapse differ from those in remission. The aim of this study was to investigate patients’ level of stress in relapse and in remission. Methods: Twenty-three patients with active IBD participated in the study. Cortisol was assessed in saliva and in blood to obtain objective measurements. For subjective measurements, the patients completed the Short Health Scale (SHS) and Perceived Stress Scale (PSS) questionnaires. Physiological measurements were taken, and questionnaires were completed at the beginning of relapse and when the patient was classified as being in remission. Relapses and remissions were determined by endoscopic examination and faecal calprotectin. Results: Cortisol levels did not differ between measurements in active disease and in remission (7.50 ± 5.52 vs. 5.55 ± 2.65). PSS showed no differences between the two measurements (13 vs. 16, *p*-value 0.83). Inflammatory cytokines IL-6 (1.97 ± 2.47 vs. 0.72 ± 0.43, *p* < 0.05) and IL-8 (6.84 ± 3.57 vs. 2.94 ± 2.79, *p* < 0.001) were significantly lower during remission compared with active disease. Conclusions: This study demonstrated low to moderate levels of perceived stress in patients with IBD during active disease and remission. The results also showed significantly elevated levels of the pro-inflammatory cytokines IL-6 and IL-8 in serum during relapse compared with remission. However, no evidence of elevated objective stress was found when levels of cortisol in saliva were measured. Further research is needed to establish the possible association between stress and IBD and how it affects patients.

## 1. Introduction

Inflammatory bowel disease (IBD) includes Crohn’s disease (CD), ulcerative colitis (UC), and indeterminate colitis. Patients are usually aged 15–35 at onset [1]. UC affects the mucosal layer in the colon, while CD can affect the entire gastrointestinal tract and all layers of the intestine [1]. IBD occurs with periods of remission and periods of relapse. Symptoms depend on the location of the inflammation, but the most common include diarrhoea, abdominal pain, rectal bleeding, and weight loss [1,2]. The cause of IBD is believed to be multifactorial, involving an interaction between genetic and environmental factors that may affect the immune system and contribute to the development of inflammation in the intestine [1,2].

Patients with IBD have increased psychological distress and lower quality of life compared to healthy individuals, especially during active disease [3,4,5,6,7,8]. Stress in its various forms may play an important role in the development and progression of IBD. However, it is not clear whether IBD causes stress and/or psychological distress like depression and anxiety or whether underlying psychological comorbidity makes individuals with IBD more prone to relapse. Stress has been shown to have a negative pathophysiological impact on gastrointestinal function and increase intestinal permeability by affecting the nervous system as well as endocrine and immunological functions [9]. The hypothalamic–pituitary–adrenal (HPA) axis is involved in the adaptive response to psychological stress, and it has been shown to play a role where neuroendocrine-immune system variations can make relapses more likely to occur [10,11]. It is still not clear whether psychological stress can be a factor for the onset of IBD, but there is increasing evidence that it may be a trigger to relapses, and many IBD patients report stress associated with a relapse [12,13,14,15].

Earlier studies have shown that high perceived stress is associated with relapses in IBD [16]. Although there are some controversies suggesting that, while high perceived stress and symptoms are associated, high perceived stress is not associated with active inflammation. Stress may enhance symptoms but not necessarily active disease [13]. Also, the nature of a temporal link is still unquantified since the time between high levels of stress and relapse has been shown to range between one day and over a year [16].

Studies have examined the effect of psychological therapies, e.g., cognitive behavioural therapy, mindfulness-based therapies like meditation and yoga, and gut-directed hypnotherapy, and found short-term improvements in stress, anxiety, and quality of life, but no improvement in disease activity or in preventing relapses [9,17,18].

Many questions remain concerning the impact of stress on IBD, such as whether patients’ level of stress is higher in a relapse compared to in remission. More evidence is also needed about a potential association between perceived stress and objectively measured stress. There is evidence that perceived stress may be associated with active disease, but no other stress subtypes [16]. The aim of this study is to investigate patients’ level of stress (both subjective and objective) in relapse and remission. With this information, we may determine if the levels of stress are higher in active disease compared to remission.

## 2. Materials and Methods

Patients diagnosed with CD or UC were recruited from Capio Gastro Center, Stockholm, Sweden. Patients were invited to participate in the study when they contacted the outpatient IBD clinic due to symptoms related to their IBD. Inclusion criteria for participants were 18 years of age or older, able to complete a questionnaire, and sufficient proficiency in the Swedish language. In addition, patients were required to have active IBD at the time of inclusion. The study was approved by the regional Ethical Review Board in Stockholm (Dnr 2016/90-31/4), and all participants provided written informed consent prior to inclusion in the study, in accordance with the Declaration of Helsinki.

### 2.1. Subjective Assessments

#### Health-Related Quality of Life (HRQOL) and Stress Questionnaires

For subjective measurements, the participants completed the Short Health Scale (SHS) and Perceived Stress Scale (PSS) questionnaires.

SHS is a disease-specific, self-administered questionnaire measuring HRQOL in IBD patients [16,17]. It comprises four items—symptom burden, social function, disease-related worry, and general well-being—with each item graded on a 6-point Likert scale. Each item has a response level scored 0–5, where higher scores indicate lower HRQOL, and the total score ranges between 0 and 20. The SHS has been translated and validated into several different languages [18,19,20,21] and can be used both in clinical and academic settings. SHS is a disease-specific, self-administered questionnaire measuring HRQOL in IBD patients [19,20]. It comprises four items—symptom burden, social function, disease-related worry, and general well-being—with each item graded on a 6-point Likert scale. Each item has a response level scored 0–5, where higher scores indicate lower HRQOL, and the total score ranges between 0 and 20. The SHS has been translated and validated into several different languages [21,22,23,24] and can be used in both clinical and academic settings.

PSS consists of ten items where the patients’ score perceived stress on a 5-point scale, 0–4 [25]. The questions concern whether the patients have felt 1—upset because of something that happened unexpectedly; 2—unable to control important things in life; 3—nervous and stressed; 4—confident with their ability to handle personal problems; 5—that things were going their way; 6—unable to cope with all the things they had to do; 7—able to control irritations in life; 8—on top of things; 9—angered because of things that happened that were outside their control; and 10—that the difficulties piled up so high that they could not be overcome [26]. The total score ranges from 0 to 40, where higher scores indicate a higher level of perceived stress. A score of 0–13 indicates a low level of stress, 14–26 indicates a moderate level of stress, and 27–40 indicates a high level of stress.

### 2.2. Objective Assessments

#### 2.2.1. Blood and Saliva Sampling

For objective measurements of stress, two sets of peripheral blood samples were taken from each patient, one during exacerbation and one during remission. Samples were drawn into sterile 10 mL tubes with a silicone-coated interior and clot activator (BD Vacutainer, East Rutherford, NJ, USA). The blood was allowed to clot for 30 min at room temperature, and then the tubes were centrifuged at 1500 *g* for 15 min. Serum was collected, aliquoted, and stored at −20 °C until subsequent laboratory analysis. Saliva samples were collected using SalivaBio Swabs during exacerbation and during remission (Salimetrics, Carlsbad, CA USA). All saliva samples were collected during daytime clinical visits (approximately between 09:00 and 15:00). Sampling times were not standardised between relapse and remission visits. Swabs were centrifuged at 1500 *g* for 15 min to release saliva from these swabs, aliquoted, and stored at −20 °C until use according to the manufacturer’s instructions.

#### 2.2.2. Serum Cytokine Analysis

Serum cytokine concentrations were quantified using the Bio-Plex Pro Human Cytokine 8-Plex assay (Bio-Rad Laboratories, Hercules, CA, USA), following the manufacturer’s instructions. The panel included granulocyte–macrophage colony-stimulating factor (GM-CSF); interferon-gamma (IFN-γ); interleukin (IL)-2, IL-4, IL-6, IL-8, IL-10; and tumour necrosis factor-alpha (TNF-α). Each sample was analysed in duplicate, and data were analysed and processed using Bio-Plex Manager software version 6.2 (Bio-Rad, Hercules, CA, USA).

#### 2.2.3. Salivary Cortisol Analysis

Cortisol was detected in saliva using salivary cortisol ELISA (enzyme-linked immunosorbent assay) (Salivary Cortisol ELISA kit, Salimetrics, State College, PA, USA), following the manufacturer’s instructions. Each sample was analysed in duplicate and quantified with a microplate reader.

#### 2.2.4. Data Management and Confidentiality

All subjective measurements (questionnaires) and collected samples (blood and saliva) were performed twice for each patient, first at the beginning of the relapse and then when the patient was classified as being in remission. Relapses and remissions were determined by endoscopic examination and faecal calprotectin. For UC patients, the Ulcerative Colitis Endoscopic Index of Severity was used, and for CD patients, the Simple Endoscopic Score for Crohn’s Disease was used. All samples and questionnaires were pseudonymised prior to analysis. The code key linking participant identity to study data was stored separately from the dataset and accessible only to the responsible researchers to ensure confidentiality.

### 2.3. Statistical Analysis

Due to the small sample size, non-parametric analyses were used and are presented as median, IQR, or proportions. The Wilcoxon Signed-Rank Test was used to compare measurements obtained from the same patients at two time points. The Mann–Whitney U-test was used to compare groups. A *p*-value less than 0.05 was considered statistically significant. Statistical analysis was performed with SPSS version 29.0.2.0., and figures were produced in GraphPad Prism version 10.6.0.

## 3. Results

### 3.1. Study Population

A total of 23 patients participated in the study, and 20 of them completed the two measurements at relapse and remission. The mean age was 44 years. Nineteen (83%) had UC, and 14 (60%) were women (Table 1). Most patients were receiving 5-aminosalicylic acid (5-ASA) as maintenance therapy at baseline. Due to relapse, some patients had their 5-ASA dose increased, while other patients were treated with cortisone or biological treatment (Table 1). The interval between the first (relapse) and second (remission) measurements was determined according to the time point at which the patient was clinically classified as being in remission. This ranged between 3 and 30 months, with a mean duration of 12 months.

### 3.2. Quality of Life Assessment

The PSS assessment categorises perceived stress into three levels: low, moderate, and high. In our study population, most participants reported a low to moderate level of perceived stress at both measurements, relapse (PSS score 13) and in remission (PSS score 16) (Table 2). The PSS scores showed no statistically significant differences between the two measurements, relapse and remission. Females reported higher scores of perceived stress compared to men at both measurements, but this was not statistically significant (Table 3). No statistically significant differences were found between patients with CD and UC, although patients with CD had higher scores of perceived stress at both measurements (Table 3). In contrast, SHS scores were significantly higher during relapse compared to remission, both for the total score and for each of the four individual domains (symptoms, function, disease-related worries, and well-being) (Table 2). This indicates an overall improvement in HRQOL during remission. Women had lower HRQOL compared to men, both at the measurement at relapse (not statistically significant) and at remission (*p* < 0.01). Patients with CD reported higher SHS scores compared to patients with UC at both measurements, although not statistically significant (Table 3).

### 3.3. Serum Cytokine Levels

**Figure 1:**
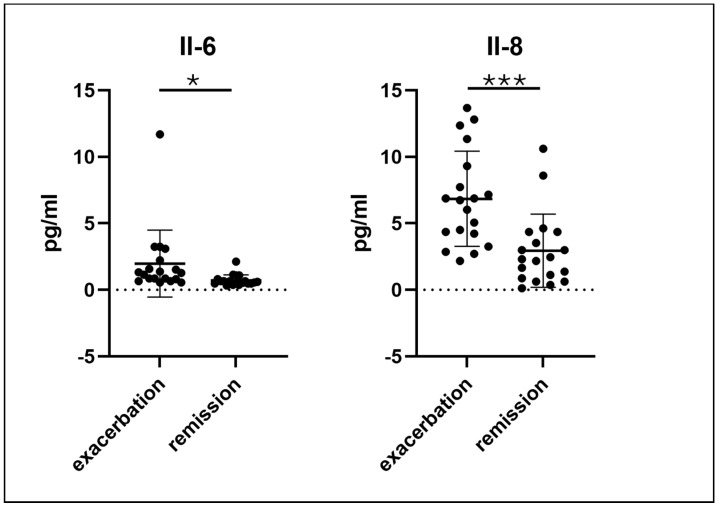
Serum cytokine levels during exacerbation (active disease) and remission in patients with IBD. Serum levels of IL-6 and IL-8 are expressed as pg/mL (mean ± SD) during exacerbation and remission. Statistical analysis was performed using a paired *t*-test comparing cytokine levels in the same patients during exacerbation and remission (* *p* < 0.05, *** *p* < 0.001).

Serum cytokine levels were analysed both during relapse and later in remission. During relapse, patients had significantly higher serum levels of IL-6 (*p* < 0.05) and IL-8 (*p* < 0.001) in comparison with values obtained during remission (Figure 1). No significant differences were observed for IFN-γ or TNF-α between the two measurements. Serum levels of GM-CSF, IL-2, IL-4, and IL-10 were below the detection limit in all samples, which may reflect very low levels of these cytokines in peripheral blood and/or limitations in assay sensitivity.

### 3.4. Saliva Cortisol Levels

The saliva cortisol levels did not differ significantly between the two assessments in relapse and remission (Table 2). 

## 4. Discussion

Cumulative evidence suggests that psychological stress is a common comorbidity in patients with IBD. However, the underlying mechanism and line of events remain unclear. This study aimed to investigate IBD patients’ levels of objective and subjective stress during active disease and remission. The results show significantly elevated levels of the pro-inflammatory cytokines IL-6 and IL-8 in serum during relapse compared with remission. These results are consistent with the well-established role of IL-6 and IL-8 in mediating intestinal inflammation and disease activity in IBD [27]. IL-6 and IL-8 elevation during relapse suggests increased immune activation, supporting their potential utility as biomarkers for disease activity [28].

Our findings correspond to previous research demonstrating that IL-6 levels correlate with clinical severity, endoscopic activity, and acute-phase inflammatory markers in IBD [29]. Similarly, elevated IL-8 has repeatedly been linked to mucosal inflammation and is often increased during active disease [30,31]. This study therefore supports the indication that both cytokines, IL-6 and IL-8, reflect inflammatory burden and may help discriminate relapse from remission.

No significant changes were observed in IFN-γ or TNF-α between relapse and remission. This corresponds with studies reporting that these cytokines may not change significantly between disease progression or may require larger sample sizes to detect differences [31]. GM-CSF, IL-2, IL-4, and IL-10 remained below detection limits in all samples, highlighting the complexity of cytokine measurement in peripheral blood, where many immune mediators are present at very low basal concentrations. This most probably reflects biologically low circulating levels of these cytokines in the study population, although limitations in assay sensitivity cannot be excluded.

IL-6 is recognised as a stress-responsive cytokine, and its elevation during active disease could reflect not only intestinal inflammation but also physiological stress associated with symptom burden [32]. However, the lack of increased cortisol levels in the same patients suggests that hypothalamic–pituitary–adrenal (HPA) axis activation was not pronounced or that chronic disease may blunt typical cortisol responses. Previous studies have shown mixed results regarding cortisol in IBD, with some reporting elevated levels during flares and others finding no difference compared with remission or controls [33]. Our results showed no significant change in saliva cortisol levels between active disease and remission, suggesting that saliva cortisol may not be a reliable standalone marker of stress in IBD populations.

It should be noted that the diurnal variation in cortisol secretion, reflected in saliva, may have influenced our results. Saliva samples were collected during daytime clinical visits, but sampling times were not standardised between relapse and remission. Since cortisol levels decline throughout the day, differences in sampling time may have masked potential differences between disease states.

The patients reported low to medium levels of perceived stress at both measurements. Interestingly, the patients perceived higher levels in remission. This could imply that even if stress is not objectively proven, the patients still experience some stress regardless of symptoms from the IBD. Targownik showed that perceived stress was associated with symptoms in IBD but no intestinal inflammation [13]. The results in this study contradict Targownik’s results, since our findings show evidence of moderate perceived stress in remission.

Bernstein et al. found higher levels of stress in patients with active disease compared to those with inactive disease [34]. However, the stress levels were stable over a three-month period, even for those patients who changed symptom status from active to inactive or from inactive to active, which corresponds with our findings.

Many patients state that stress may trigger a relapse in IBD, and several studies also show evidence on the association between perceived stress and activity in IBD [12,14,15,34,35,36,37,38]. However, evidence is lacking on the nature of this link. Earlier studies vary considerably in terms of the time from stress to relapse in IBD (from 1 day to 18 months). It is not yet clarified if perceived stress may trigger a relapse or if the relapse may trigger stress. Little is known about the association between stress and an actual relapse rather than only intestinal symptoms. The reduced inflammation in the intestine may be the result of increased or modified pharmacological treatment when patients were admitted with relapse. Anti-inflammatory treatment affects IL6 and IL8 but not necessarily stress levels [39].

While pharmacological treatment addresses the underlying mucosal inflammation, it does not necessarily resolve the associated psychological or neurophysiological mechanisms, such as stress responses and visceral hypersensitivity, which may contribute to persistent symptoms. This study measures stress during relapse and then remission in the same patient, with the aim of examining any difference between those two periods. No differences were found either in subjective or objective stress between active and inactive disease. The results, however, showed a discrepancy between subjective and objective measures of stress. While no elevated cortisol levels were observed, patients reported moderate levels of perceived stress during both active disease and remission.

Stress clearly has a major impact on IBD, and several studies have shown beneficial effects of different psychological interventions on stress levels, which also lead to improved HRQOL [40,41]. A systematic review showed that mindfulness-based interventions resulted in improvements in stress, both in the short and long term [42]. Another systematic review showed that psychological therapies had valuable short-term effects on stress, anxiety, depression, and HRQOL, but no effect in improving disease activity or in preventing relapse in IBD [17]. A randomised controlled study with patients from Norway and Germany examined the effect of a psychological programme [43]. Each of the participants had experienced a relapse during the past 18 months and high chronic stress levels. The patients in the intervention group were offered a combination of psychoeducation treatments, which consisted of instruction in structured problem solving and relaxation training in combination with cognitive behavioural therapy-based techniques. The intervention did not improve the IBD course and did not reduce relapses but showed an improvement in HRQOL in UC patients (but not in CD patients). Both groups had decreased and normal values of perceived stress after 18 months, but there were no differences between the groups [43]. Even if stress reduction may not decrease the direct risk of relapse in IBD, it may provide other benefits that can have an indirect effect on the disease. In this study, the levels of perceived stress remained medium-high, while the scores of SHS improved when the patients came into remission.

Psychosocial factors, such as social network, financial, and occupational status, have been shown to influence HRQOL and perceived stress [44]. In this study, such factors were not examined, and therefore, no conclusions concerning this can be drawn. Also, the length of time between relapse and remission varied a lot between the participants (3–30 months), which can mean that several other factors may have influenced the disease course. However, in this study, no measurements were made for the treatment regimen after baseline.

Only 20 patients completed the study. However, to our knowledge, few studies have examined both objective and subjective stress in the same patients during active and inactive disease. Also, regarding the small sample size and overrepresentation of UC patients, subgroup analyses should be interpreted with caution.

Overall, our findings highlight the complex relationship between inflammation and stress responses in IBD. While inflammatory markers were elevated during relapse, perceived stress remained relatively stable regardless of disease activity, and no differences were observed in cortisol levels. Considering the limited sample size and variability in follow-up time, this study may contribute to the understanding of subjective and objective stress in patients with IBD during relapse and remission.

## 5. Conclusions

This study could show medium levels of perceived stress both in active and inactive disease in patients with IBD. However, there was no evidence of abnormal levels of objective stress when this was measured by estimating cortisol in saliva. The SHS scores showed improvements when the patients were in remission compared to active disease. Further research is needed to establish the possible association between stress and IBD and how it affects the patients. Further research should include both subjective and objective measurements.

## Figures and Tables

**Table 1:** Background data of the included patients (*n* = 23).

Sex, *n* (%)	
Female	14 (60)
Male	9 (40)
Diagnosis, *n* (%)	
UC	19 (83)
CD	4 (17)
Age, mean (SD)	44 (14.5)
Maintenance medical treatment at inclusion, *n* (%)	
5-ASA monotherapy	15 (66)
Thiopurine monotherapy	3 (13)
Thiopurine and 5-ASA	1 (4)
Biological and 5-ASA	1 (4)
Biological and Thiopurine	1 (4)
No treatment	2 (9)
Prescribed medical treatment due to relapse, *n* (%)	
5-ASA dose increased	8 (35)
Topical 5-ASA	1 (4)
Cortisone	8 (35)
Biological treatment initiated or increased dose	6 (26)
Smoking, *n* (%)	
Smoker	2 (9)
Non-smoker	12 (52)
Former smoker	9 (39)

**Table 2:** Results at baseline (active disease) and follow-up (remission).

	Baseline	IQR	Follow-Up	IQR	*p*-Value
Cortisol in saliva, nmol/L	7.50 ± 5.52		5.55 ± 2.65		
PSS, Total median score (range 0–40)	13	13	16	13	0.83
SHS, Total mean score (range 0–20)	9	5	4	6	**<0.001**
SHS: Symptoms (range 0–5)	3	2	0	2	**<0.001**
SHS: Function (range 0–5)	2	2	1	2	**<0.001**
SHS: Anxiety (range 0–5)	2	1	1	2	**<0.001**
SHS: General well-being (range 0–5)	2	1	1	1	0.08

Significant values are presented in bold.

**Table 3:** Comparisons between gender and diagnosis in PSS and SHS.

	Baseline	*p*-Value	Follow-Up	*p*-Value
PSS, total median score				
Male/Female	11/19	0.05	11/14	0.05
CD/UC	15/12	0.92	19/11	0.10
SHS, Total median score				
Male/Female	7/10	0.05	2/6	**0.01**
CD/UC	10/8	0.23	6/3.5	0.24

Significant values are presented in bold.

## Data Availability

The research data can be accessed by contacting susanna.jaghult@ki.se.

## Abbreviations

The following abbreviations are used in this manuscript:

IBD	Inflammatory bowel disease
UC	Ulcerative colitis
CD	Crohn’s disease
HRQOL	Health-related quality of life

## References

[1] Seyedian S.S., Nokhostin F., Malamir M.D. (2019). A review of the diagnosis, prevention, and treatment methods of inflammatory bowel disease. J. Med. Life.

[2] Flynn S., Eisenstein S. (2019). Inflammatory Bowel Disease Presentation and Diagnosis. Surg. Clin. N. Am..

[3] Knowles S.R., Graff L.A., Wilding H., Hewitt C., Keefer L., Mikocka-Walus A. (2018). Quality of Life in Inflammatory Bowel Disease: A Systematic Review and Meta-analyses-Part I. Inflamm. Bowel Dis..

[4] Gracie D.J., Williams C.J., Sood R., Mumtaz S., Bholah M.H., Hamilin P.J., Ford A.C. (2016). Poor Correlation Between Clinical Disease Activity and Mucosal Inflammation, and the Role of Psychological Comorbidity, in Inflammatory Bowel Disease. Am. J. Gastroenterol..

[5] Leone D., Gilardi D., Corro B.E., Menichetti J., Vegni E., Correale C., Mariangela A., Furfaro F., Bonovas S., Peyrin-Biroulet L. (2019). Psychological Characteristics of Inflammatory Bowel Disease Patients: A Comparison Between Active and Nonactive Patients. Inflamm. Bowel Dis..

[6] Barberio B., Zamani M., Black C.J., Savarino E.V., Ford A.C. (2021). Prevalence of symptoms of anxiety and depression in patients with inflammatory bowel disease: A systematic review and meta-analysis. Lancet Gastroenterol. Hepatol..

[7] Knowles S.R., Wilson J.L., Connell W.R., Kamm M.A. (2011). Preliminary examination of the relations between disease activity, illness perceptions, coping strategies, and psychological morbidity in Crohn’s disease guided by the common sense model of illness. Inflamm. Bowel Dis..

[8] Lonnfors S., Vermeire S., Greco M., Hommes D., Bell C., Avedano L. (2014). IBD and health-related quality of life—Discovering the true impact. J. Crohns Colitis.

[9] Rozich J.J., Holmer A., Singh S. (2020). Effect of Lifestyle Factors on Outcomes in Patients with Inflammatory Bowel Diseases. Am. J. Gastroenterol..

[10] Labanski A., Langhorst J., Engler H., Elsenbruch S. (2020). Stress and the brain-gut axis in functional and chronic-inflammatory gastrointestinal diseases: A transdisciplinary challenge. Psychoneuroendocrinology.

[11] Tache Y., Larauche M., Yuan P.Q., Million M. (2018). Brain and Gut CRF Signaling: Biological Actions and Role in the Gastrointestinal Tract. Curr. Mol. Pharmacol..

[12] Sexton K.A., Walker J.R., Graff L.A., Bernstein M.T., Beatie B., Miller N., Sargent M., Targownik L.E. (2017). Evidence of Bidirectional Associations Between Perceived Stress and Symptom Activity: A Prospective Longitudinal Investigation in Inflammatory Bowel Disease. Inflamm. Bowel Dis..

[13] Targownik L.E., Sexton K.A., Bernstein M.T., Beatie B., Sargent M., Walker J.R., Graff L.A. (2015). The Relationship Among Perceived Stress, Symptoms, and Inflammation in Persons with Inflammatory Bowel Disease. Am. J. Gastroenterol..

[14] Bernstein C.N., Singh S., Graff L.A., Walker J.R., Miller N., Cheang M. (2010). A prospective population-based study of triggers of symptomatic flares in IBD. Am. J. Gastroenterol..

[15] Jäghult S., Saboonchi F., Möller J., Johansson U.B., Wredling R., Kapraali M. (2013). Stress as a Trigger for Relapses in IBD: A Case-Crossover Study. Gastroenterol. Res..

[16] Black J., Sweeney L., Yuan Y., Singh H., Norton C., Czuber-Dochan W. (2022). Systematic review: The role of psychological stress in inflammatory bowel disease. Aliment. Pharmacol. Ther..

[17] Riggott C., Mikocka-Walus A., Gracie D.J., Ford A.C. (2023). Efficacy of psychological therapies in people with inflammatory bowel disease: A systematic review and meta-analysis. Lancet Gastroenterol. Hepatol..

[18] Bernabeu P., van-der Hofstadt C., Rodriguez-Marin J., Gutierrez A., Alonso M.R., Zapater P., Jover R., Sempere L. (2021). Effectiveness of a Multicomponent Group Psychological Intervention Program in Patients with Inflammatory Bowel Disease: A Randomized Trial. Int. J. Environ. Res. Public Health.

[19] Stjernman H., Granno C., Järnerot G., Ockander L., Tysk C., Blomberg B., Strom M., Hjortswang H. (2008). Short health scale: A valid, reliable, and responsive instrument for subjective health assessment in Crohn’s disease. Inflamm. Bowel Dis..

[20] Hjortswang H., Jarnerot G., Curman B., Sandberg-Gertzen H., Tysk C., Blomberg B., Almer S., Strom M. (2006). The Short Health Scale: A valid measure of subjective health in ulcerative colitis. Scand. J. Gastroenterol..

[21] Demmer S., Kleindienst N., Hjortswang H., Thomann P., Ebert M., Reindl W., Thomann A. (2023). Validation of the German version of the Short Health Scale—A brief, valid and reliable instrument to assess health-related quality of life in German-speaking patients with inflammatory bowel diseases. Z. Gastroenterol..

[22] Jelsness-Jorgensen L.P., Bernklev T., Moum B. (2012). Quality of life in patients with inflammatory bowel disease: Translation, validity, reliability and sensitivity to change of the Norwegian version of the short health scale (SHS). Qual. Life Res..

[23] Coenen S., Weyts E., Geens P., Vermeire S., Ferrante M., Vanhaecht K., Van Assche G. (2019). Short Health Scale: A valid and reliable measure of quality of life in Dutch speaking patients with inflammatory bowel disease. Scand. J. Gastroenterol..

[24] McDermott E., Keegan D., Byrne K., Doherty G.A., Mulcahy H.E. (2013). The Short Health Scale: A valid and reliable measure of health related quality of life in English speaking inflammatory bowel disease patients. J. Crohns Colitis.

[25] Sewitch M.J., Abrahamowicz M., Bitton A., Daly D., Wild G.E., Cohen A., Katz S., Szego P.L., Dobkin P.L. (2001). Psychological distress, social support, and disease activity in patients with inflammatory bowel disease. Am. J. Gastroenterol..

[26] Burt C.E., Cohen L.H., Bjorck J.P. (1988). Perceived family environment as a moderator of young adolescents’ life stress adjustment. Am. J. Community Psychol..

[27] Cibor D., Szczeklik K., Koziol K., Pocztar H., Mach T., Owczarek D. (2020). Serum concentration of selected biochemical markers of endothelial dysfunction and inflammation in patients with the varying activity of inflammatory bowel disease. Pol. Arch. Intern. Med..

[28] Nowak J.K., Kalla R., Satsangi J. (2023). Current and emerging biomarkers for ulcerative colitis. Expert. Rev. Mol. Diagn..

[29] Godala M., Gaszynska E., Walczak K., Malecka-Wojciesko E. (2023). Role of Serum Interleukin-6, Interleukin-1beta and Interleukin-10 in Assessment of Disease Activity and Nutritional Status in Patients with Inflammatory Bowel Disease. J. Clin. Med..

[30] Keshavarzian A., Fusunyan R.D., Jacyno M., Winship D., MacDermott R.P., Sanderson I.R. (1999). Increased interleukin-8 (IL-8) in rectal dialysate from patients with ulcerative colitis: Evidence for a biological role for IL-8 in inflammation of the colon. Am. J. Gastroenterol..

[31] Bourgonje A.R., von Martels J.Z.H., Gabriels R.Y., Blokzijl T., Buist-Homan M., Heegsma J., Jansen B.H., van Dullemen H.M., Festen E.A.M., Ter Steege R.W.F. (2019). A Combined Set of Four Serum Inflammatory Biomarkers Reliably Predicts Endoscopic Disease Activity in Inflammatory Bowel Disease. Front. Med..

[32] Gao X., Cao Q., Cheng Y., Zhao D., Wang Z., Yang H., Wu Q., You L., Wang Y., Lin Y. (2018). Chronic stress promotes colitis by disturbing the gut microbiota and triggering immune system response. Proc. Natl. Acad. Sci. USA.

[33] Brzozowski B., Mazur-Bialy A., Pajdo R., Kwiecien S., Bilski J., Zwolinska-Wcislo M., Mach T., Brzozowski T. (2016). Mechanisms by which Stress Affects the Experimental and Clinical Inflammatory Bowel Disease (IBD): Role of Brain-Gut Axis. Curr. Neuropharmacol..

[34] Bernstein M.T., Targownik L.E., Sexton K.A., Graff L.A., Miller N., Walker J.R. (2016). Assessing the Relationship between Sources of Stress and Symptom Changes among Persons with IBD over Time: A Prospective Study. Can. J. Gastroenterol. Hepatol..

[35] Camara R.J., Schoepfer A.M., Pittet V., Begre S., von Kanel R. (2011). Mood and nonmood components of perceived stress and exacerbation of Crohn’s disease. Inflamm. Bowel Dis..

[36] Hirten R.P., Danieletto M., Scheel R., Shervey M., Ji J., Hu L., Sauk J., Chang L., Arnrich B., Bӧttinger E. (2021). Longitudinal Autonomic Nervous System Measures Correlate with Stress and Ulcerative Colitis Disease Activity and Predict Flare. Inflamm. Bowel Dis..

[37] Langhorst J., Hofstetter A., Wolfe F., Hauser W. (2013). Short-term stress, but not mucosal healing nor depression was predictive for the risk of relapse in patients with ulcerative colitis: A prospective 12-month follow-up study. Inflamm. Bowel Dis..

[38] Wintjens D.S.J., de Jong M.J., van der Meulen-de Jong A.E., Romberg-Camps M.J., Becx M.C., Maljaars J.P., van Bodegraven A.A., Mahmmod N., Markus T., Haans J. (2019). Novel Perceived Stress and Life Events Precede Flares of Inflammatory Bowel Disease: A Prospective 12-Month Follow-Up Study. J. Crohns Colitis.

[39] Reinisch W., Sandborn W.J., Hommes D.W., D’Haens G., Hanauer S., Schreiber S., Panaccione R., Fedorak R.N., Tighe M.B., Huang B. (2011). Adalimumab for induction of clinical remission in moderately to severely active ulcerative colitis: Results of a randomised controlled trial. Gut.

[40] Wynne B., McHugh L., Gao W., Keegan D., Byrne K., Rowan C., Hartery K., Kirschbaum C., Doherty G., Cullen G. (2019). Acceptance and Commitment Therapy Reduces Psychological Stress in Patients with Inflammatory Bowel Diseases. Gastroenterology.

[41] Schlee C., Uecker C., Oznur O., Bauer N., Langhorst J. (2024). Participants’ perspectives on a multimodal stress management and comprehensive lifestyle modification program for patients with Crohn’s disease—A qualitative interview study. PLoS ONE.

[42] Ewais T., Begun J., Kenny M., Rickett K., Hay K., Ajilchi B., Kisely S. (2019). A systematic review and meta-analysis of mindfulness based interventions and yoga in inflammatory bowel disease. J. Psychosom. Res..

[43] Boye B., Lundin K.E., Jantschek G., Leganger S., Mokleby K., Tangen T., Jantschek I., Pripp A.H., Wojniusz S., Dahlstroem A. (2011). INSPIRE study: Does stress management improve the course of inflammatory bowel disease and disease-specific quality of life in distressed patients with ulcerative colitis or Crohn’s disease? A randomized controlled trial. Inflamm. Bowel Dis..

[44] Moradkhani A., Beckman L.J., Tabibian J.H. (2013). Health-related quality of life in inflammatory bowel disease: Psychosocial, clinical, socioeconomic, and demographic predictors. J. Crohns Colitis.

